# Surgical outcomes and prognoses of patients with clinical stage I lung cancer and interstitial lung disease

**DOI:** 10.1007/s11748-025-02240-0

**Published:** 2025-12-29

**Authors:** Hidenao Kayawake, Momoko Soda, Masakazu Takayama, Yuhei Yokoyama, Tetsu Yamada, Ryo Tachikawa, Keisuke Tomii, Hiroshi Hamakawa, Yutaka Takahashi

**Affiliations:** 1 Department of Thoracic Surgery, Kobe City Medical Center General Hospital, Kobe, Japan; 2 Department of Thoracic Surgery, Kyoto University, 54 Shogoin-Kawahara-cho, Sakyo-ku, Kyoto, 606-8507 Japan; 3 Department of Respiratory Medicine, Kobe City Medical Center General Hospital, Kobe, Japan

**Keywords:** Interstitial lung disease, Clinical stage I lung cancer, Acute exacerbation, Second primary lung cancer occurrence, Prognosis

## Abstract

**Objective:**

Studies have reported poor surgical outcomes in patients with lung cancer and interstitial lung disease. Therefore, we retrospectively analyzed the perioperative and long-term outcomes of this patient population.

**Methods:**

Between 2004 and 2021, we enrolled 103 patients with interstitial lung disease and clinical stage I lung cancer (8th edition of the TNM classification) without a history of lung cancer treatment within 5 years before surgery and undergoing complete resection from our institution.

**Results:**

The median patient age was 74 years (range: 60–89 years), and 90 patients were male. The most common surgical procedures were lobectomy (n = 85), followed by partial resection (n = 13), segmentectomy (n = 4), and pneumonectomy (n = 1). The median observation period was 1102 days. Concerning perioperative outcomes, 90-day postoperative mortality was 7 (6.8%) and complications (≥ Grade 3 according to the Clavien-Dindo classification) were observed in 30 patients (29.1%). Regarding long-term outcomes, lung cancer recurrence was observed in 38 patients. Fifty-six patients died during the observation period, but only 20 (35.7%) died of lung cancer recurrence. Pathological upstaging was observed in 51 patients (49.5%). The 5-year overall survival and recurrence-free survival rates were 48.6% and 41.8%, respectively.

**Conclusions:**

A relatively high 90-day mortality rate was observed. Deaths from causes other than lung cancer recurrence were observed more frequently than those from lung cancer recurrence. Hence, when selecting treatment strategies for early lung cancer combined with interstitial lung disease, the risks of acute exacerbation and progression of interstitial lung disease should be considered.

**Supplementary Information:**

The online version contains supplementary material available at 10.1007/s11748-025-02240-0.

## Introduction

Lung cancer is a leading cause of cancer-related death worldwide [1]. Generally, lung cancer occurs more frequently in patients with interstitial lung disease (ILD) than in those without [2, 3]. The frequency of lung cancer occurrence in patients with ILD is approximately four times higher than that in patients without ILD [4], and the lung cancer occurrence rate is approximately 5–15% among patients with ILD [5, 6]. Although surgical resection is the main treatment option for early stage lung cancer, acute exacerbation (AE) of ILD (AE-ILD) is a concern for patients with lung cancer and ILD. Previously, the occurrence rate of AE-ILD was reported as about 5–15% postoperatively [7–9], and approximately half of the patients experiencing AE-ILD postoperatively died early after surgical resection [7, 8]. Moreover, some studies indicate that the prognoses of patients with lung cancer and ILD are worse than those of patients without ILD [10, 11]; however, few studies have reported whether worse prognoses are derived from lung cancer or other factors. Therefore, we retrospectively analyzed the perioperative and long-term postoperative outcomes of patients with clinical stage I (cStage I) lung cancer and clinically diagnosed ILD.

## Patients and methods

Between 2004 and 2021, 2317 lung cancer surgeries were performed at our institution. Among them, 176 were clinically diagnosed with ILD by a pulmonologist. This study included 103 patients with stage I lung cancer and ILD who underwent R0 or R1 resection without a history of lung cancer treatment within 5 years before surgery. Perioperative and long-term outcomes of these 103 patients were retrospectively analyzed. The diagnoses of ILD are comprehensively made based on preoperative image findings and postoperative pathological findings. Idiopathic pulmonary fibrosis (IPF) is basically diagnosed after multidisciplinary discussion according to the IPF guidelines [12, 13].

The perioperative outcomes, postoperative hospital days, duration of postoperative drainage, occurrence rates of postoperative complications (≥ grade 3 Clavien-Dindo) [14, 15], postoperative AE-ILD, and 90-day mortality rate were investigated. Regarding long-term outcomes, we investigated the actuarial overall survival (OS), recurrence-free survival (RFS), causes of death, and occurrence rate of secondary primary lung cancer. Both clinical and pathological staging were performed based on the 8th edition of the tumor node metastasis (TNM) classification of lung cancer [16]. This study was approved by the Institutional Review Board of Kobe City Medical Center General Hospital (approval number zn240202). The requirement for informed consent was waived because of the retrospective nature of the study.

### Statistical analysis

Descriptive statistics were obtained using the EZR software, a graphical user interface for R (The R Foundation for Statistical Computing, Vienna, Austria) [17, 18]. Continuous variables are presented as medians with ranges, and categorical variables are expressed as percentages. The observation period was calculated based on the number of postoperative months to the patient’s last follow-up or death. The observation period for RFS was calculated based on the number of postoperative months to last follow-up, cancer recurrence, or death. The follow-up was censored at the end of 2023. The median observation period was 1102 days (range, 14-5070 days). Actuarial survival rates were calculated using the Kaplan–Meier method, and groups were compared using a log-rank test. Statistical significance was defined as p-value of less than 0.05.

## Results

### Patient characteristics

Patient characteristics are shown in Table 1. The median patient age was 74 years (range: 60–89 years), and 90 patients (87.4%) were male. The median Brinkman index was 1000 (range: 0–3600), and the variety of performance status (PS) of the patients was as follows; PS 0 in 74 patients (71.8%), PS 1 in 28 patients (27.2%) and PS 2 in one patient (1.0%). The histological results for lung cancer included squamous cell carcinoma (n = 55, 53.4%), adenocarcinoma (n = 36, 35.0%), small cell carcinoma (n = 3, 2.9%), pleomorphic carcinoma (n = 2, 1.9%), and others (n = 7, 6.8%). According to the TNM classification of lung cancer, the preoperative clinical stages of non-small cell lung cancer (NSCLC) were stage IA1 (n = 5, 4.9%), IA2 (n = 40, 38.8%), IA3 (n = 29, 28.2%), and IB (n = 29, 28.2%). Thirty-eight patients (36.9%) were clinically diagnosed with IPF according to the IPF guidelines [17, 18]. The median interstitial pneumonia (IP) risk score [19] was 7 (range: 3–20), and 103 patients were divided into low-risk (n = 75, 72.8%), intermediate-risk (n = 25, 24.3%), and high-risk (n = 3, 2.9%) groups. The primary surgical resection was lobectomy (n = 85, 82.5%), followed by partial resection (n = 13, 12.6%), and segmentectomy (n = 4, 3.9%). IP risk stratification did not significantly influence the operative methods. Lobectomy was mainly performed in high risk (n = 3, 100%), in intermediate risk (n = 22, 88%), and in low risk (80%). All 103 patients underwent R0 resection. Although the prevention of AE-ILD by the administration of anti-fibrotic agents has not basically been performed in our institution, the administration of pirfenidone was performed in 12 cases (11.6%) and that of nintedanib was done in one case (1.0%). On the other hand, preoperative steroid usage was observed in 9 cases (8.7%).

**Table 1:** Patient characteristics

Variables	Number (%) or median (range)
Age (years)	74.0 (60–89)
*Sex*	
Male	90 (87.4%)
Female	13 (12.6%)
Body mass index (kg/m2)	23.2 (16.2–28.3)
Brinkman index	1000 (0-3600)
Charlson comorbidity index	2.0 (0–8)
*Performance status (PS)*	
PS 0	74 (71.8%)
PS 1	28 (27.2%)
PS 2	1 (1.0%)
*Lung cancer histology*	
Squamous cell carcinoma	55 (53.4%)
Adenocarcinoma	36 (35.0%)
Small cell carcinoma	3 (2.9%)
Pleomorphic carcinoma	2 (1.9%)
Others	7 (6.8%)
*Clinical stage*	
IA1	5 (4.9%)
IA2	40 (38.8%)
IA3	29 (28.2%)
IB	29 (28.2%)
*Diagnosis of ILD*	
IPF	38 (36.9%)
Indeterminate or non-IPF	65 (63.1%)
IP risk score	7 (3–20)
*IP risk stratification*	
Low-risk group	75 (72.8%)
Intermediate-risk group	25 (24.3%)
High-risk group	3 (2.9%)
Preoperative antifibrotic therapy	13 (12.6%)
Pirfenidone	12 (11.6%)
Nintedanib	1 (1.0%)
Preoperative steroid usage	9 (8.7%)
Radiologically UIP pattern	31 (30.1%)
History of AE-ILD	4 (3.9%)
KL-6 > 1000	12 (11.7%)
%VC ≤ 80	19 (18.4%)
*Operative methods*	
Lobectomy	85 (82.5%)
Pneumonectomy	1 (1.0%)
Segmentectomy	4 (3.9%)
Partial resection	13 (12.6%)
*Residual tumor status*	
R0	103 (100.0%)

PS, performance status; ILD, interstitial lung disease; IPF, idiopathic pulmonary fibrosis; IP, interstitial pneumonia; UIP, usual interstitial pneumonia; AE-ILD, acute exacerbation of ILD; VC, vital capacity

## Perioperative outcomes

Perioperative outcomes are outlined in Table 2. The median duration of postoperative drainage was 2 days (range: 1–59 days), and the median postoperative hospital stay was 5 days (range: 2–61 days). Postoperative complications ( ≧ Grade 3a) were observed in 30 patients (29.1%). The most frequent postoperative complication ( ≧ Grade 3a) was air leak (n = 15, 50%), followed by postoperative AE-ILD (n = 6, 20%) and empyema (n = 3, 10.0%). The 30-day mortality rate was 2.9% (n = 3), and the 90-day mortality rate was 6.8% (n = 7). The six patients who experienced postoperative AE-ILD comprised three low-risk and three intermediate-risk patients. All seven patients who died within 90 days postoperatively underwent lobectomy (Table 3). The causes of 90-day mortality included AE-ILD (n = 5), acute heart failure (n = 1), and unknown causes (n = 1). The distribution of the IP risk score was as follows: two patients were classified as low-risk, four as intermediate-risk, and one as high-risk.

**Table 2:** Perioperative outcomes

Variables	Number (%) or median (range)
Postoperative hospital stay (days)	5.0 (2–61)
Postoperative drainage (days)	2.0 (1–59)
*Postoperative complication (≥ Grade 3)*	
Yes	30 (29.1%)
No	73 (70.9%)
*Details of postoperative complication (≥ Grade 3)*	
Air leak	15 (50.0%)
AE-ILD	6 (20.0%)
Empyema	3 (10.0%)
Atelectasis	2 (6.7%)
Others	4 (13.3%)
30-day mortality	3 (2.9%)
90-day mortality	7 (6.8%)

AE-ILD, acute exacerbation of interstitial lung disease

**Table 3:** Case series of the seven patients who died within 90 days postoperatively

Case	Age	Sex	BMI (kg/㎡)	PS	Brinkman index	Charlson comorbidity index	%VC	cStage	IP risk score	IP risk stratification	Operation	Operation time (min)	pStage	Cause of death
1	60	M	20.5	1	1200	1	111.2	IA2	11	Intermediate-risk	Open lobectomy	195	IA3	AE-ILD
2	79	M	19.3	1	1200	0	84.9	IA3	11	Intermediate-risk	Open lobectomy	185	IA3	AE-ILD
3	75	F	23.9	0	0	1	117.4	IA3	8	Low-risk	VATS lobectomy	215	IA2	AE-ILD
4	65	M	26.5	0	1500	1	79.5	IA2	12	Intermediate-risk	VATS lobectomy	333	IA2	AE-ILD
5	79	M	22.4	1	2400	2	46.9	IB	15	High-risk	VATS lobectomy	101	IB	Unknown
6	75	M	23.1	1	1000	2	62.4	IB	8	Low-risk	VATS lobectomy	351	IIIA	Acute heart failure
7	76	M	22.9	1	1140	7	78.2	IA2	12	Intermediate-risk	VATS lobectomy	113	IA3	AE-ILD

BMI, body mass index; PS, performance status; VC, vital capacity; cStage, clinical stage; IP, interstitial pneumonia; pStage, pathological stage; AE-ILD, acute exacerbation of interstitial lung disease

## Long-term outcomes and prognoses

Postoperative recurrence was observed in 38 (36.9%) patients during the study period (Table 4). During the observation period, 56 (54.4%) patients died. Deaths from lung cancer recurrence was observed in 20 patients (35.7%), whereas those from other causes, including chronic respiratory failure and AE-ILD occurring in the chronic phase, were observed in 31 patients (55.4%), indicating that deaths from other causes were higher than those from lung cancer recurrence. Secondary primary lung cancer occurred in 13 patients (12.6%) .

**Table 4:** Patient long-term outcomes

Variables	Number (%)
*Postoperative recurrence*	
Yes	38 (36.9%)
No	65 (63.1%)
*Deaths during the observation period*	
Yes	56 (54.4%)
No	47 (45.6%)
Second primary lung cancer occurrence	13 (12.6%)
*Causes of deaths*	
Deaths from lung cancer recurrence	20 (35.7%)
Deaths associated with surgery (hospital death)	5 (8.9%)
Others (including respiratory failure and acute exacerbation of ILD)	31 (55.4%)

**Fig. 1:**
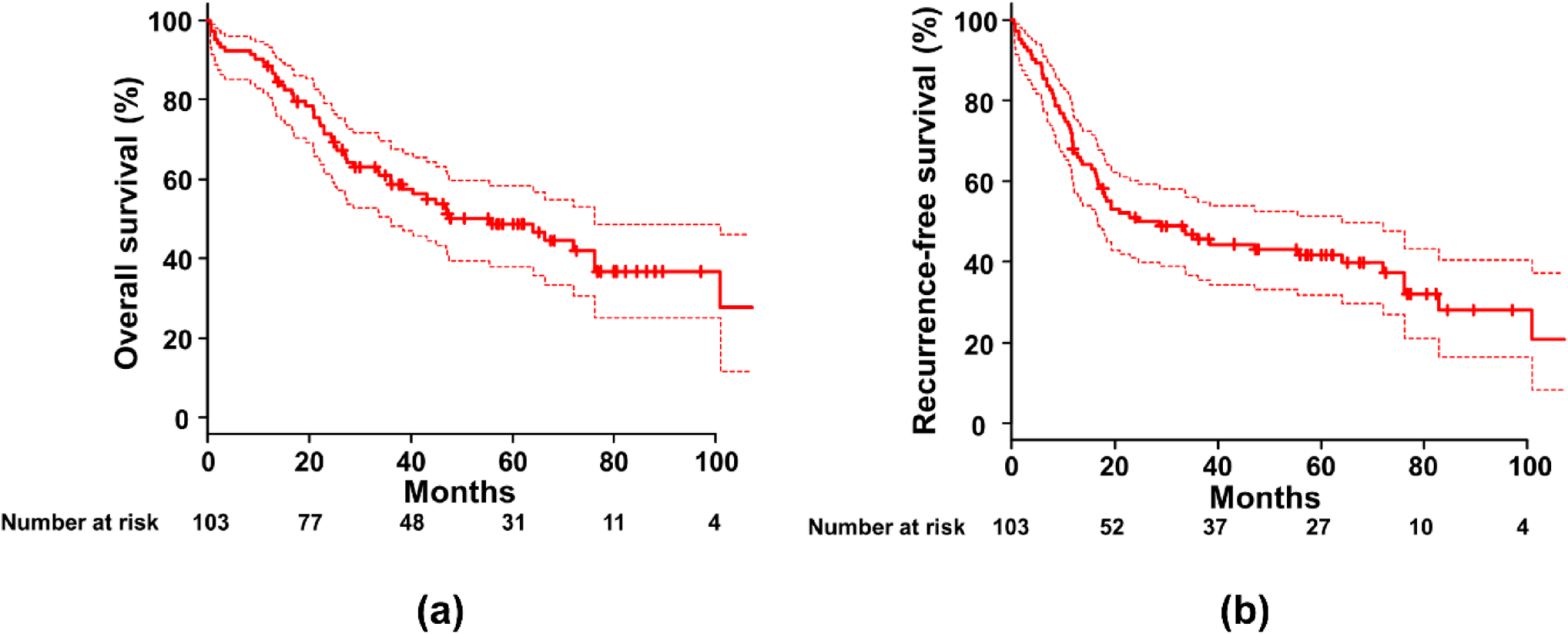
Survival rates of the study cohort **a** The OS of all 103 patients is shown here. The 5-year OS was only 48.6% (95% CI: 38.0–58.4%). **b** The 5-year RFS was 41.8% (95% CI: 31.8–51.4%) OS, overall survival; cStage, clinical stage; CI, confidence interval; RFS, recurrence-free survival

**Fig. 2:**
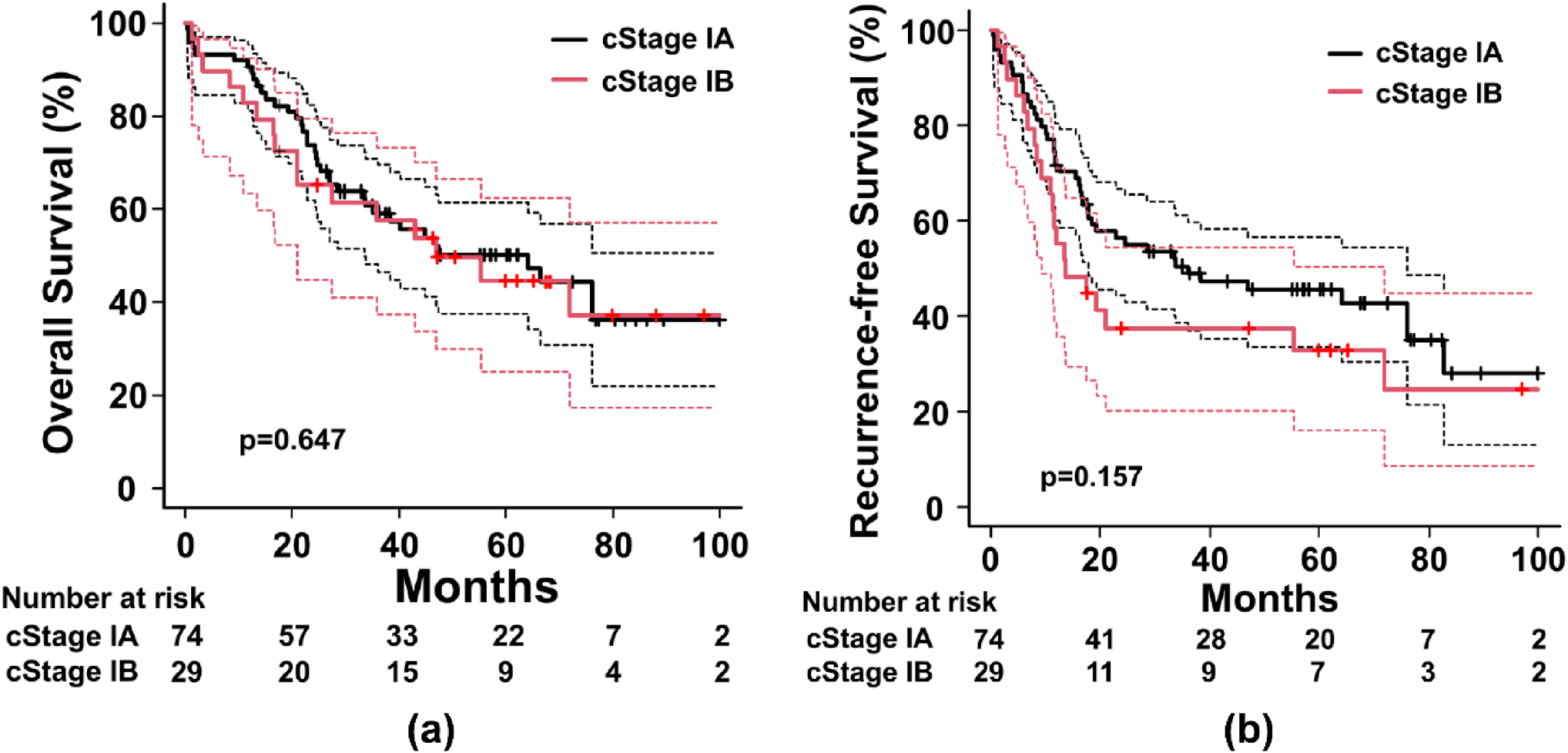
Survival rates of the study cohort according to cStage **a** The OS was compared between patients with cStage IA lung cancer and cStage IB lung cancer. No significant difference was observed in OS between the two groups (*p* = 0.647). The 5-year OS of patients with cStage IA lung cancer was 50.2% (95% CI: 37.5–61.5%), while that of patients with cStage IB lung cancer was 44.6% (95% CI: 25.1–62.3%). **b** Regarding RFS, the RFS of patients with cStage IA lung cancer was slightly better than that of those with cStage IB lung cancer; however, the difference did not reach significance (*p* = 0.157). The 5-year RFS was 45.4% (95% CI: 33.5–56.6%) for patients with cStage IA and 32.7% (95% CI: 16.2–50.3%) for those with cStage IB OS, overall survival; cStage, clinical stage; CI, confidence interval; RFS, recurrence-free survival

The 5-year OS of the entire cohort was 48.6% (95% confidence interval [CI]: 38.0–58.4%, Fig. 1A). Similarly, the 5-year RFS rate was only 41.8% (95% CI: 31.8–51.4%, Fig. 1B). There was no significant difference in OS between patients with cStage IA (n = 74) or cStage IB (n = 29) lung cancer (*p* = 0.647, Fig. 2A). The 5-year OS of patients with cStage IA lung cancer was 50.2% (95% CI: 37.5–61.5%), while that of cStage IB was 44.6% (95% CI: 25.1–62.3%). Moreover, the RFS of patients with cStage IA lung cancer was slightly better than that of cStage IB; however, the difference was not statistically significant (*p* = 0.157, Fig. 2B).The 5-year RFS was 45.4% (95% CI: 33.5–56.6%) for patients with cStage IA lung cancer and 32.7% (95% CI: 16.2–50.3%) for those with cStage IB.

### Stage migration

One of the reasons for the poor prognosis in this study seemed to be stage migration. In the present study, pathological upstaging was observed in 51 patients (49.5%). Upstaging was mainly derived from T factors (n = 39, 76.5%); however, in six cases (11.8%), the N factor was related, and in the remaining six cases, both T and N factors were related. Among the 45 cases in which the T factor was upgraded, the main reason for T factor upgrading was a size increase in pathology (n = 24, 53.3%), followed by visceral pleural invasion in pathology (n = 17, 37.8%). For reference, prognoses based on pathological stage were calculated (Supplemental Fig. 1A and 1B). The 5-year OS was as follows; 53.0% (95% CI: 38.1–65.8) in p-Stage 0-IA, 48.0% (95% CI: 26.8–66.4%) in p-Stage IB, 53.8% (95% CI: 24.8–76.0%) in p-Stage II and 22.9% (95% CI: 3.5–52.2%) in p-Stage III. The 5-year RFS was 47.3% (95% CI: 33.3–60.1%) in p-Stage 0-IA, 40.6% (95% CI: 20.8–59.6%) in p-Stage IB, 38.5% (95% CI: 14.1–62.8%) in p-Stage II, and 20.0% (95% CI: 3.1–47.5%) in p-Stage III, respectively.

## Subgroup analysis

As a subgroup analysis, we compared the postoperative outcomes between patients having and not having IPF. Regarding the OS, it was not significantly different (the 5-year OS: 45.8% [95% CI: 29.1–61.0%] vs. 50.3% [95% CI: 36.6–62.6%], *p* = 0.352, Supplemental Fig. 2A). Similarly, RFS was not significantly different (the 5-year RFS: 46.4% [95% CI: 29.8–61.4%] vs. 39.1% [95% CI: 26.8–51.2%], *p* = 0.73, Supplemental Fig. 2B). Regarding the long-term outcomes, the frequency of death from lung cancer among IPF patients was slightly lower than that among indeterminate or non-IPF patients, but the difference did not reach the statistical significance (29.2% vs. 40.6%, *p* = 0.335).

As another subgroup analysis, we divided the patients into two groups (Early period [patients undergoing surgical resection between 2004 and 2014, n = 46] and Late period [patients undergoing surgical resection between 2015 and 2021, n = 57]) based on the number of patients included. It was observed that the OS among patients of the Late period was non-significantly better than that of the Early period (the 5-year OS: 58.3% [95% CI: 43.5–70.4%] vs. 37.9% [95% CI: 23.5–52.2%], *p* = 0.073, Supplemental Fig. 3A). Similarly, the RFS among patients of the Late period was non-significantly better than that of the Early period (the 5-year RFS: 51.2% [95% CI: 37.3–63.6%] vs. 31.1% [95% CI: 18.2–45.0%], *p* = 0.07, Supplemental Fig. 3B). Thirty-day mortality was observed only in the Early period; however, 90-day mortality was observed both in the Early (n = 4) and the Late period (n = 3).

## Discussion

The present study yielded several important findings. First, the 90-day mortality rate was relatively high and the occurrence rate of postoperative AE-ILD was 5.8%. Second, all seven patients who died within 90 days after surgery underwent lobectomy. Third, deaths from causes other than lung cancer recurrence occurred more frequently than deaths from lung cancer recurrence. Finally, second primary lung cancer was observed in 12.6% of patients; hence, close follow-up may be warranted for these patients.

In a previous nationwide Japanese study, the occurrence rate of postoperative AE-ILD was 9.3%, and the 30-day surgical mortality was 2.6% [7]. From this surveillance, the operative risks of patients with IP were evaluated, resulting in an IP risk calculator [19]. In this study, although the 90-day mortality rate was 6.8%, which was considered relatively high, since the occurrence rate of postoperative AE-ILD was 5.8% and the majority of patients who experienced postoperative AE-ILD died within 90 days postoperatively, our results were considered reasonable.

As one of the risk factors for postoperative AE-ILD, anatomical resection including, segmentectomy and lobectomy, were detected in the IP risk calculator [19]. Regarding the operative method used in this study, the main surgery was lobectomy, and all patients who died within 90 days postoperatively underwent lobectomy. In contrast, more than 70% of the patients enrolled in this study were evaluated as low risk, and half of the patients who experienced postoperative AE-ILD were preoperatively evaluated as low risk. In fact, the majority of patients who died within 90 days or experienced postoperative AE-ILD underwent surgery in the first half of the study period; therefore, advances in surgical procedures and perioperative management may have influenced the results.

Regarding prognoses, the 5-year OS was only 48.6% and the 5-year RFS was 41.8% in this study, which are very poor prognoses for cStage I lung cancer. These results are comparable to those of previous single-institution studies in Japan [10, 11], indicating that our results are reasonable. Although lung cancer recurrence was observed in 38 patients (36.9%), our results showed that the frequency of deaths from causes other than lung cancer recurrence was higher than that from lung cancer recurrence, suggesting that more attention should be paid to ILD as well as lung cancer. A previous multi-institutional Japanese study indicated that deaths from lung cancer were more frequent than deaths from other causes [2]. Recent single-institutional studies have reported that deaths from lung cancer were more frequently observed than deaths from other causes [11], while another report showed that the number of deaths from causes other than lung cancer was higher than that from lung cancer recurrence [3]. Therefore, whether death from lung cancer recurrence or from other causes is more frequently observed remains unclear. This study indicated that the number of deaths from causes other than lung cancer recurrence was higher, which may be partly influenced by the fact that approximately one-third of the patients enrolled in this study had IPF. ILD, especially IPF, has been known for poor prognoses. Previously,* the median survival of adult IPF patients was reported to be 3.8 years* [20].

In lung cancer combined with ILD, precise preoperative staging has often been reported to be difficult, partly because there are some cases in which the preoperative tumor size calculated from imaging modalities is not concordant with the postoperative pathological tumor size and because lymph node enlargements are sometimes observed in these patients due to the inflammation derived from ILD [21, 22]. This study showed that upstaging of pathological findings was observed in approximately half of the patients, which was considered one of the reasons why the prognoses were poor for cStage I lung cancer. The majority of the surgical procedures in this study were lobectomies. Previously, the long-term outcomes of patients who underwent wedge resection were reported to be poorer than for those undergoing lobectomy [2]. Our results regarding frequent pathological upstaging indicated that it was difficult to perform wedge resection while securing adequate surgical margins for lung cancer combined with ILD, which could be one of the reasons for the poor prognosis associated with wedge resection in previous studies.

The prevalence of lung cancer among patients with ILD is calculated as 10–15% [6, 23]. The second primary lung cancer occurrence rate was 12.6% in this study, indicating that a close follow-up to check the presence or absence of a second primary lung cancer is necessary for these patients, as well as to evaluate whether lung cancer recurrence occurs. Furthermore, second primary lung cancer occurrence is considered to result in worse prognoses because the treatment strategies for second primary lung cancer are limited due to poor pulmonary function after lung resection for primary lung cancer.

 Regarding the histology of lung cancer combined with ILD, squamous cell carcinoma was most frequently observed in this study. This result was compatible with previous studies indicating that the frequency of squamous cell carcinoma is higher than adenocarcinoma among lung cancer patients having ILD [24, 25].

This study had a few limitations. First, this was a single-center, retrospective, non-randomized study. Therefore, multicenter studies with larger cohorts are required to validate our findings. Second, this study included patients who underwent surgery over a long study period; therefore, the surgical procedures and postoperative management may have changed over time. However, a strength of our study is that few patients were lost to follow-up because patients with lung cancer combined with ILD were followed up in cooperation with pulmonologists.

## Conclusion

As deaths from causes other than lung cancer recurrence were observed more frequently than those from lung cancer recurrence, the selection of treatment strategies for lung cancer combined with ILD should consider the risks of AE-ILD and the progression of ILD itself. Furthermore, close follow-up may be warranted for these patients because of the occasional occurrence of a second primary lung cancer.

## Supplementary Information

Below is the link to the electronic supplementary material.

Supplementary Material 1: Survival rates of the study cohort according to pStage(a) The 5-year OS was 53.0% (95% CI: 38.1-65.8) in p-Stage 0-IA, 48.0% (95% CI: 26.8-66.4%) in p-Stage IB, 53.8% (95% CI: 24.8-76.0%) in p-Stage II and 22.9% (95% CI: 3.5-52.2%) in p-Stage III, respectively. (b) The 5-year RFS was as follows; 47.3% (95% CI: 33.3-60.1%) in p-Stage 0-IA, 40.6% (95% CI: 20.8-59.6%) in p-Stage IB, 38.5% (95% CI: 14.1-62.8%) in p-Stage II, and 20.0% (95% CI: 3.1-47.5%) in p-Stage III.

Supplementary Material 2: Survival rates of the study cohort comparing between patients having and not having IPF(a) Regarding the OS, it was not significantly different between patients having and not having IPF (the 5-year OS: 45.8% [95% CI: 29.1-61.0%] vs 50.3% [95% CI: 36.6-62.6%], p=0.352). (b) Similarly, RFS was not significantly different between the two groups (the 5-year RFS: 46.4% [95% CI: 29.8-61.4%] vs 39.1% [95% CI: 26.8-51.2%], p=0.73).

Supplementary Material 3:Survival rates of the study cohort comparing Early period to Late period(a) It was observed that the OS among patients of the Late period was non-significantly better than that of the Early period (the 5-year OS: 58.3% [95% CI: 43.5-70.4%] vs 37.9% [95% CI: 23.5-52.2%], p=0.073). (b) Similarly, the RFS among patients of the Late period was non-significantly better than that of the Early period (the 5-year RFS: 51.2% [95% CI: 37.3-63.6%] vs 31.1% [95% CI: 18.2-45.0%], p=0.07).Survival rates of the study cohort comparing Early period to Late period(a) It was observed that the OS among patients of the Late period was non-significantly better than that of the Early period (the 5-year OS: 58.3% [95% CI: 43.5-70.4%] vs 37.9% [95% CI: 23.5-52.2%], p=0.073). (b) Similarly, the RFS among patients of the Late period was non-significantly better than that of the Early period (the 5-year RFS: 51.2% [95% CI: 37.3-63.6%] vs 31.1% [95% CI: 18.2-45.0%], p=0.07).
