# Supplementary figures and images for: Surgical outcomes and prognoses of patients with clinical stage I lung cancer and interstitial lung disease
Source: Gen Thorac Cardiovasc Surg. 2025 Dec 29;74(5):549–56. doi: 10.1007/s11748-025-02240-0 (PMC13139283; doi:10.1007/s11748-025-02240-0)

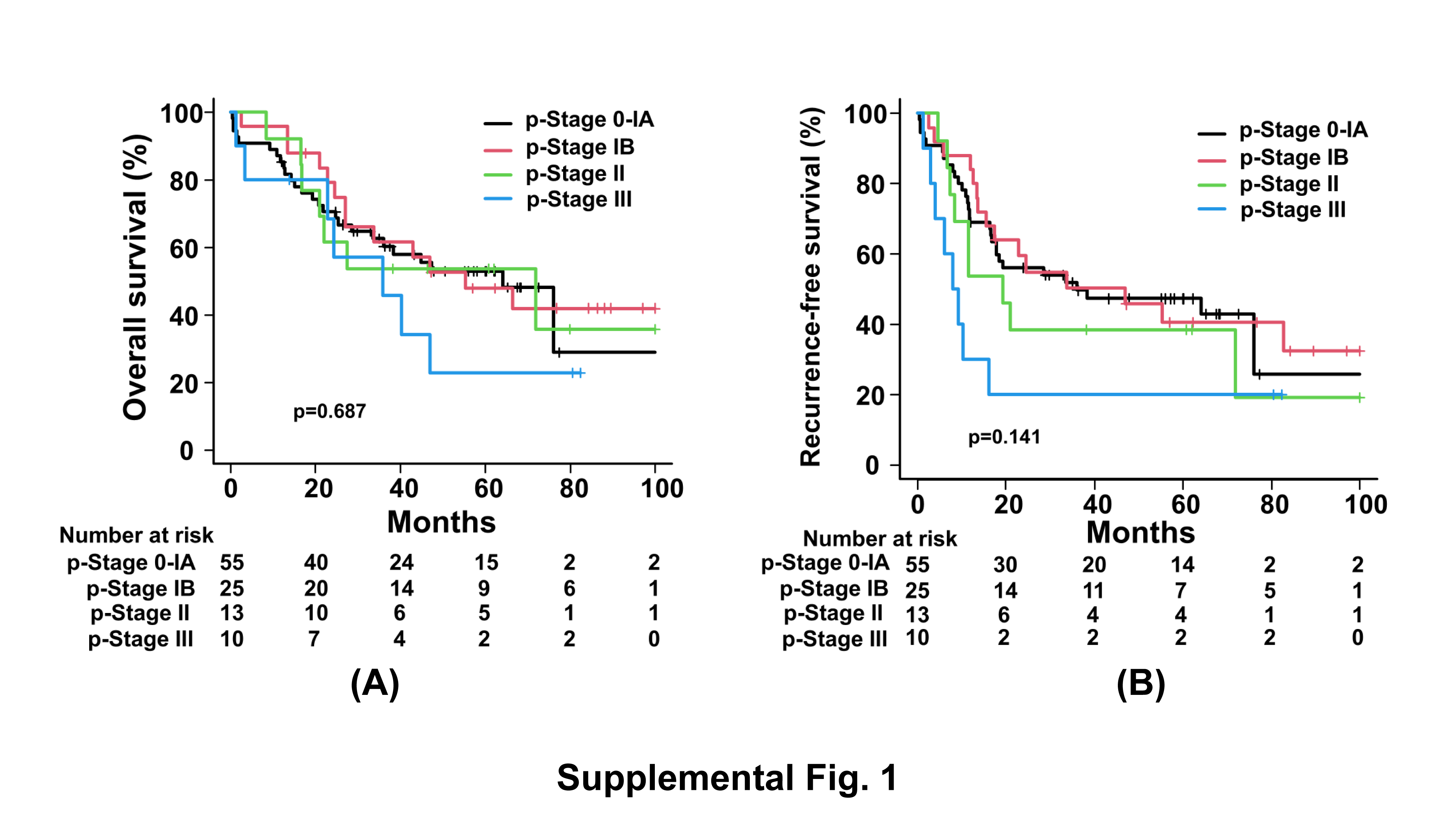

Supplement: Supplementary file 1 — Supplementary Material 1: Survival rates of the study cohort according to pStage(a) The 5-year OS was 53.0% (95% CI: 38.1-65.8) in p-Stage 0-IA, 48.0% (95% CI: 26.8-66.4%) in p-Stage IB, 53.8% (95% CI: 24.8-76.0%) in p-Stage II and 22.9% (95% CI: 3.5-52.2%) in p-Stage III, respectively. (b) The 5-year RFS was as follows; 47.3% (95% CI: 33.3-60.1%) in p-Stage 0-IA, 40.6% (95% CI: 20.8-59.6%) in p-Stage IB, 38.5% (95% CI: 14.1-62.8%) in p-Stage II, and 20.0% (95% CI: 3.1-47.5%) in p-Stage III. [file 11748_2025_2240_MOESM1_ESM.tif]

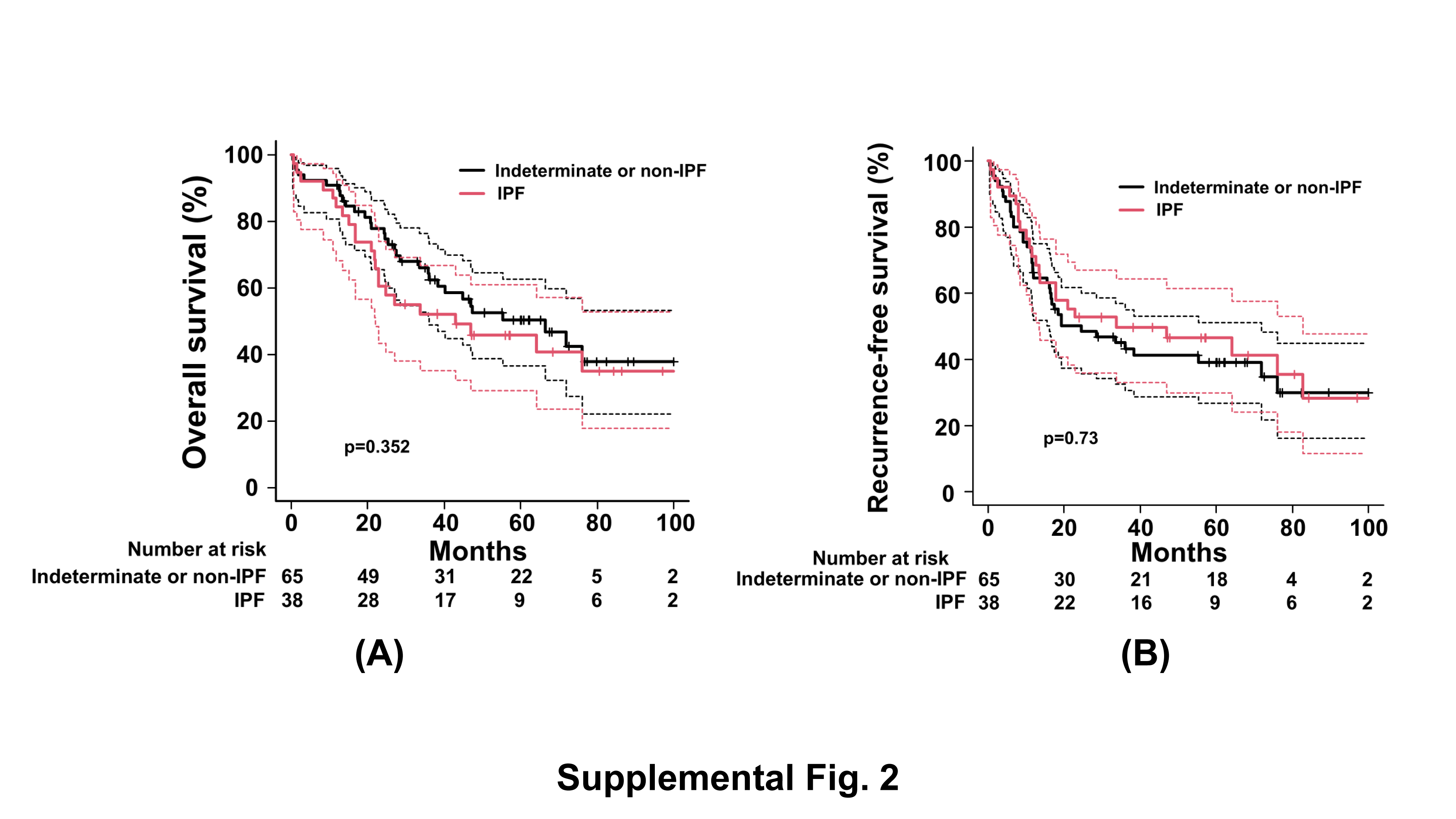

Supplement: Supplementary file 2 — Supplementary Material 2: Survival rates of the study cohort comparing between patients having and not having IPF(a) Regarding the OS, it was not significantly different between patients having and not having IPF (the 5-year OS: 45.8% [95% CI: 29.1-61.0%] vs 50.3% [95% CI: 36.6-62.6%], p=0.352). (b) Similarly, RFS was not significantly different between the two groups (the 5-year RFS: 46.4% [95% CI: 29.8-61.4%] vs 39.1% [95% CI: 26.8-51.2%], p=0.73). [file 11748_2025_2240_MOESM2_ESM.tif]

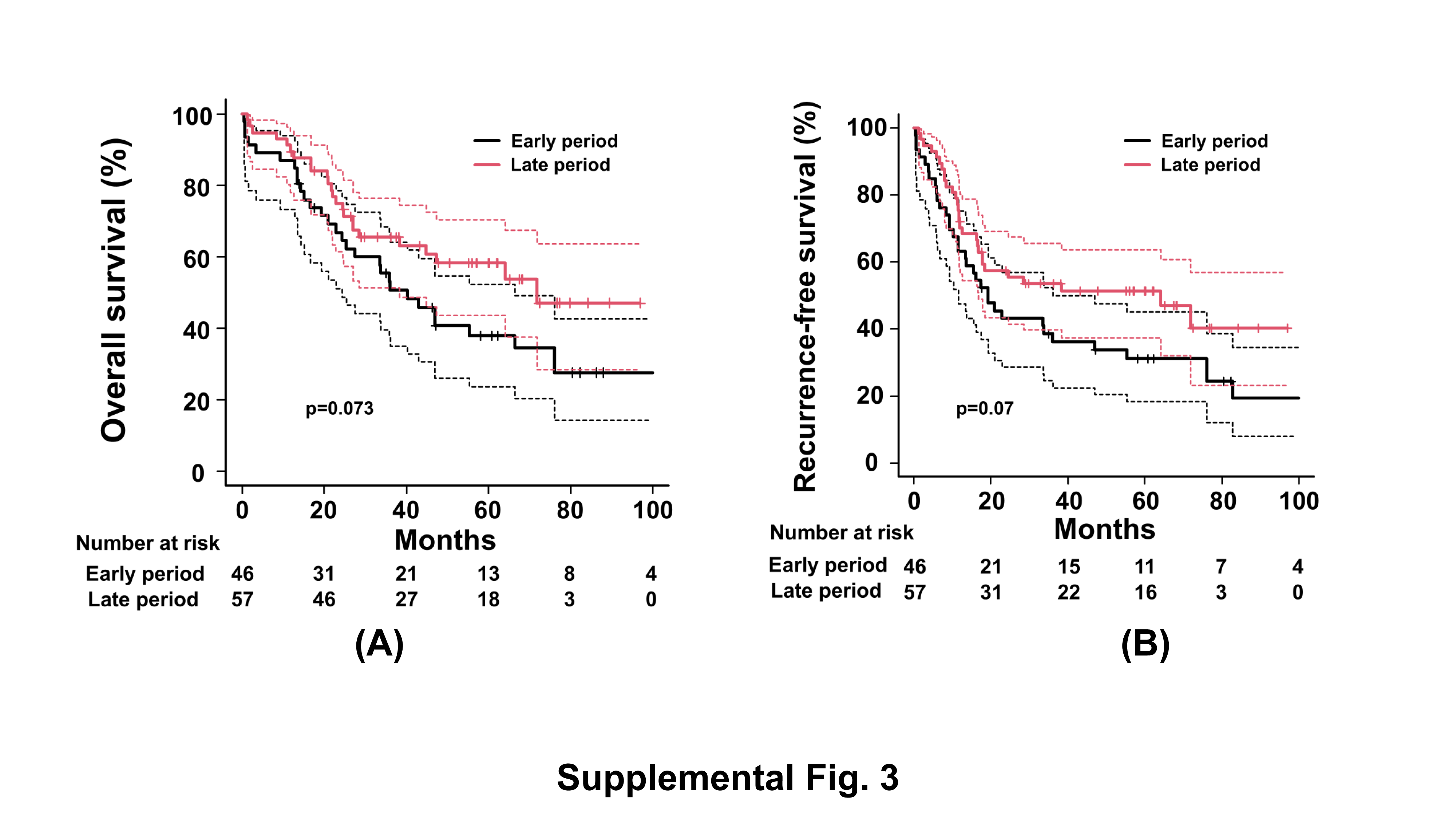

Supplement: Supplementary file 3 — Supplementary Material 3:Survival rates of the study cohort comparing Early period to Late period(a) It was observed that the OS among patients of the Late period was non-significantly better than that of the Early period (the 5-year OS: 58.3% [95% CI: 43.5-70.4%] vs 37.9% [95% CI: 23.5-52.2%], p=0.073). (b) Similarly, the RFS among patients of the Late period was non-significantly better than that of the Early period (the 5-year RFS: 51.2% [95% CI: 37.3-63.6%] vs 31.1% [95% CI: 18.2-45.0%], p=0.07).Survival rates of the study cohort comparing Early period to Late period(a) It was observed that the OS among patients of the Late period was non-significantly better than that of the Early period (the 5-year OS: 58.3% [95% CI: 43.5-70.4%] vs 37.9% [95% CI: 23.5-52.2%], p=0.073). (b) Similarly, the RFS among patients of the Late period was non-significantly better than that of the Early period (the 5-year RFS: 51.2% [95% CI: 37.3-63.6%] vs 31.1% [95% CI: 18.2-45.0%], p=0.07). [file 11748_2025_2240_MOESM3_ESM.tif]
